# Premature mortality from respiratory disease attributable to PM_2.5_ exposure in Lanzhou, Gansu Province, China

**DOI:** 10.13075/ijomeh.1896.02721

**Published:** 2026

**Authors:** Yipakezi Aiken, Lingqing Wang, Xiaoning Liu

**Affiliations:** 1 Lanzhou University, Department of Health Statistics and Epidemiology, School of Public Health, Lanzhou, China; 2 Lanzhou Centre for Disease Control and Prevention, Department of Health Statistics and Epidemiology, School of Public Health, Lanzhou, China

**Keywords:** PM_2.5_, Lanzhou, elderly population, spatiotemporal distribution, premature mortality, GEMM

## Abstract

**Objectives:**

Exposure to particulate matter 2.5 (PM_2.5_) remains a critical public health issue in China. Understanding its spatial distribution and associated mortality burden is crucial for developing effective preventive strategies.

**Material and Methods:**

This study analyzed PM_2.5_ distribution and estimated premature mortality from chronic obstructive pulmonary disease (COPD) and lung cancer attributable to PM_2.5_. The analysis covered different districts and 3 age groups in Lanzhou City, using the global exposure mortality model (GEMM).

**Results:**

From 2014 to 2023, PM_2.5_ concentrations were significantly reduced city-wide. However, the number of PM_2.5_-attributable premature deaths did not decline substantially. In some districts, these deaths even increased against a backdrop of rising overall mortality. The majority of premature deaths occurred in people aged ≥65 years. Lowering PM_2.5_ concentrations remains important for controlling mortality from both diseases. However, the trends were not entirely consistent, indicating a complex relationship between PM_2.5_ and mortality.

**Conclusions:**

Particulate matter 2.5 continues to have a substantial impact on health. Sustained efforts in air quality improvement and targeted health interventions for the elderly population are necessary.

## Highlights

- Particulate matter 2.5 (PM_2.5_) fell in Lanzhou, China, yet premature respiratory deaths did not decline.
- Aging and baseline mortality rises offset air quality improvements.
- Global exposure mortality model used to estimate district-level PM_2.5_-attributable deaths.
- Elderly and urban districts bear higher PM_2.5_ health burdens.
- Targeted policies needed for chronic obstructive pulmonary disease and lung cancer disparities.

## INTRODUCTION

Globally, about 6.67 million premature deaths were due to air pollution in 2019. Particulate matter pollution is listed as the fifth leading cause of death on the Global Burden of Disease (GBD) study [1]. Fine particulate matter with aerodynamic diameter ≤2.5 μm (PM_2.5_) is the most widely studied among all air pollutants with its harmful impacts on human health all over the world, according to the latest GBD 2021, exposure to ambient PM_2.5_ resulted in 4.83 million deaths worldwide in 2021. The number of global deaths attributable to ambient PM_2.5_ is estimated to have increased from 3.5 million in 1990 to 4.83 million in 2021 [2]. Despite prematurely achieving its 2030 target of a 55% reduction in PM_2.5_ related health impacts (from 2005 levels) with a 57% decrease by 2023, the European Union (EU) still recorded 182 000 premature deaths attributable to PM_2.5_ in that year [3].

With the industrialization and rapid economic development in the past decades, PM_2.5_ pollution has become a leading environmental challenge in China [4]. To tackle the severe PM_2.5_ pollution, the government has implemented several control policies. In 2013, China issued the Air Pollution Prevention and Control Action Plan (APPCAP) which improved PM_2.5_ concentration [5]. In addition, due to rapid urbanization and improved economic conditions, the rural-urban migrant population has switched to cleaner fuel types, contributing significantly to the decline in PM_2.5_ concentrations in China. However, the GBD analysis found that ambient PM_2.5_ pollution resulted in approx. 1.4 million premature deaths in 2019 in China [1].

According to the World Health Organization (WHO) report on Sustainable Development Goal Indicator 3.9.1, in 2019, an estimated 1 034 497 deaths from chronic obstructive pulmonary disease (COPD) and 352 520 deaths from lung cancer were attributable to the joint effects of ambient and household air pollution. These figures represent approx. 30% of all global COPD deaths and 11% of all global lung cancer deaths in that year [6], highlighting air pollution as a significant and preventable risk factor for these respiratory diseases. A direct comparison of these population-attributable fractions reveals a substantially higher burden for COPD than for lung cancer. Among the diseases of the respiratory system, COPD is the leading cause of death in the world [7]. Notably, among the numerous health outcomes linked to PM_2.5_, chronic respiratory diseases are the leading causes of mortality and morbidity worldwide, and COPD leads to the most deaths [8]. This aligns with global exposure-response evidence indicating that a 10 μg/m^3^ increase in PM_2.5_ levels was associated with an 18% increase in the incidence of COPD [9]. Regarding another major outcome, lung cancer was also the leading cause of cancer death, with an estimated 1.8 million deaths (18.7%) as reported by the International Agency for Research on Cancer (IARC) in 185 countries [10].

The global trends described above are critically reflected in the Chinese context. In China, it is thought that there were significant adverse effects of PM_2.5_ on the incidence rates of lung cancer for both males and females [11]. Lung cancer, the most common malignancy in China, is also the leading cause of cancer-related mortality [12]. This alignment between global patterns and the national situation underscores the universality of the challenge.

Beyond the epidemiological associations, the adverse health impacts of PM_2.5_, including both chronic disease mechanisms and acute effects [13,14], are grounded in well-characterized biological pathways relevant to COPD and lung cancer. PM_2.5_ can cause oxidative stress, which is triggered by the catalyzation of biochemical reactions, the activation of oxidases and metabolic enzymes, and mitochondrial dysfunction, all of which can lead to lung injury and aggravate various respiratory diseases including COPD and lung cancer [15], besides these, chronic inflammation and epigenetic alterations are also important mechanisms [16,17]. Through these mechanisms, PM_2.5_ exerts varying degrees of acute and chronic effects on human health.

This study focused on Lanzhou city, the capital of Gansu Province, northwest China. It is located in the convergence of the Loess Plateau, Qinghai-Tibet Plateau, and Inner Mongolia Plateau, making it prone to sand and dust storms [18]. The industrial structure of the city was dominated by heavy industry such as petrochemical, metallurgical, and mechanical industries, and there is a 5-month-long heating period in winter every year. According to local source apportionment studies, PM_2.5_ in Lanzhou primarily originates from multiple sources including soil dust, coal combustion, industrial and vehicle emissions, secondary sulfate, and biomass burning, with secondary aerosols, coal combustion, and vehicle emissions being identified as the dominant contributors [19]. During the process of urban development, the emission of PM_2.5_ exceeds the atmospheric self-purification capacity. Before the implementation of the air pollution prevention and control plan, the air quality in Lanzhou City was poor and ranked among the top 10 severely air-polluted cities in the country. After comprehensive management, the air quality of Lanzhou City has improved significantly [20].

The authors' previous study analyzed that the crude mortality rate of the population in Lanzhou City has a rising tendency from 2014–2021 and is higher than that at the national level. The neoplasms and diseases of the respiratory system was ranked the second and third causes of death, respectively. Lung cancer is the first cause of death among all cancers, and COPD is the top cause of death among diseases of the respiratory system in 2014–2021 in all ages. These 2 diseases of the respiratory system are related to air pollutants. Thus, this study aimed to clarify the relationship between PM_2.5_ health risks and population death caused by COPD and lung cancer. On the basis of 2014 (before the implementation of the plan) and 2023 (after the implementation of the plan) PM_2.5_ monitoring data and the resident death database, global exposure mortality model (GEMM) was used to estimate the premature mortality of COPD and lung cancer due to PM_2.5_ at the district/county level. And to explore the changes in the concentration of PM_2.5_ and the impact of PM_2.5_ on the health of the population.

## MATERIAL AND METHODS

### Data source

The annual average concentration of PM_2.5_ for each district/county of Lanzhou City, China, is calculated based on the yearly PM_2.5_ raster data at 1 km resolution nationwide in 2000–2023, released by the National Tibetan Plateau Science Data Center, based on the administrative boundary data. The death surveillance data and population data were collected from the cause of death registration and reporting information system of the Lanzhou Center for Disease Control and Prevention. The PM_2.5_ concentration, cause-of-death data, and demographic data used in this study are all from the years 2014 and 2021.

### Estimation of PM_2.5_ concentration in Lanzhou city

The population density was used to weight and calculate the average PM_2.5_ concentration for the entire city of Lanzhou, from which the average premature mortality rate was calculated.


(1) $$C_{\text{weighted}}=\frac{\sum_{i=1}^{n}\left(C_{i}\times D_{i}\right)}{\sum_{i=1}^{n}D_{i}}$$

where:

C_i_ – the PM_2.5_ concentration of each district,

D_i_ – the population density of each district.

### Calculation of PM_2.5_-attributed death

Burnett et al. [21] found that long-term exposure to PM_2.5_ was closely related to premature deaths caused by ischemic heart disease (IHD), cerebrovascular disease (CEV) (stroke), COPD, lung cancer (LC), and lower respiratory infections (LRI). Based on a large amount of data from 41 cohorts in 16 countries, an innovative GEMM was established to calculate premature deaths attributable to PM_2.5_. The GEMM assumes a logarithmic relationship between exposure and baseline risk ratios and incorporates results from a Chinese cohort study [21]. Chinese scholars proved that the GEMM results were in better agreement with census-based estimation [22].

This study focused on COPD and LC. The relationship between PM_2.5_ concentrations and mortality are described by the following hazard ratio (HR) functions:

(2) HR(z)=exp{θT(z)}

where:

θ – the concentration-response model coefficient,

z – observed PM_2.5_ concentration 2.4 µg/m^3^,

T(z) – the complex transformation function applied to the concentration.

T(z) is calculated as follows:

(3) T(z)=f(z)ω(z)

(4) f(z)=log(1+z/α)

(5) ω(z)=1/(1+exp{−(z−μ)/(v)})

where:

α, μ, ν – the curved form of the hazard ratio function.

Overall, through specifying the parameters (α, μ, ν) in Table 1, the HR(z) can be calculated [21].


(6) $$M=y_{0}\times \mathrm{Pop}\times \left(\frac{\mathrm{HR}-1}{\mathrm{HR}}\right)$$

where:

M – premature mortality caused by PM_2.5_ exposure,

y_0_ – disease-specific baseline mortality rates,

Pop – the adult population (>25 years of age) exposed to PM_2.5_ in a certain area.

**Table 1.** Global exposure mortality model (GEMM) parameter estimates for the population >25 years by cause of death

Cause of death	θ	SE(θ)	α	μ	ν
Chronic obstructive pulmonary disease	0.2510	0.6762	6.5	2.5	32
Lung cancer	0.2942	0.06147	6.2	9.3	29.8

Considering the uncertainty of HR in the model, a 95% confidence interval (CI) was calculated using the standard error [SE(θ)] in the GEMM.

In this study, the authors considered the resident population as the exposed population for their calculations.

### Simulation of mortality of reduced PM_2.5_ concentrations

To quantify the association between PM_2.5_ concentration and premature mortality under current conditions, several scenarios were established. Assuming a gradual reduction in PM_2.5_ from the level of 57.38 μg/m³, the authors calculated the corresponding premature mortality rate and its 95% CI for each concentration. The concentration threshold for a statistically significant decrease in PM_2.5_-attributable deaths was identified.

### Model stability assessment

To assess the stability of their primary model, the authors conducted a validation analysis using the integrated exposure-response (IER) model [23]. This alternative approach yielded estimates for PM_2.5_-attributable mortality from lung cancer and COPD. The overall trends, geographical patterns, and magnitude of the disease burden estimated by the IER model were consistent with those derived from the primary GEMM model. While point estimates differed as expected due to variations in the concentration-response functions, the direction and significance of the association between PM_2.5_ exposure and attributable mortality remained robust. This consistency across 2 independent and widely used risk models strengthens confidence in the stability of the authors' primary findings and suggests that the conclusions are not highly sensitive to the choice of a specific concentration-response function. Detailed data from the stability analysis are provided in the results.

### Ethics statement

This research involved the analysis of modelled exposure estimates and publicly reported health statistics. As the study did not involve human participants, animal subjects, or primary data collection, ethical approval was not required.

## RESULTS

### Spatiotemporal characteristics of PM_2.5_ in 2014 and 2023

**Figure 1.**
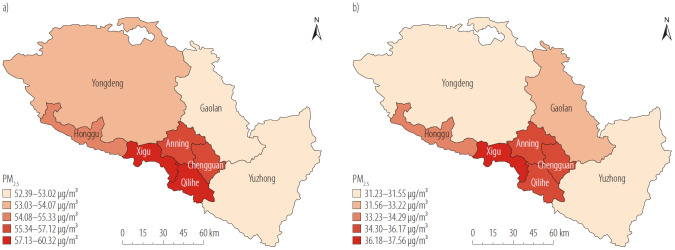
Spatial distribution of particulate matter with aerodynamic diameter ≤2.5 μm (PM_2.5_) in Lanzhou city, China, in a) 2014 and b) 2023

Lanzhou City consists of 3 counties and 5 districts. The average concentrations of PM_2.5_ is significantly decreased from 57.38 μg/m^3^ in 2014 to 36.08 μg/m^3^ in 2023. In 2014, PM_2.5_ concentrations were generally at high levels, with all districts failing to meet Grade II (35 μg/m^3^) of China's National Ambient Air Quality Standards (GB 3095-2012). In 2023, the 4 main urban areas Chengguan district (36.17 μg/m^3^), Qilihe district (35.54 μg/m^3^), Xigu district (37.56 μg/m^3^) and Anning district (36.21 μg/m^3^) were not meeting the national Grade II standards, but the 3 counties where Yongdeng county (31.23 μg/m^3^), Gaolan county (33.22 μg/m^3^) and Yuzhong county (31.55 μg/m^3^) and Honggu district (34.29 μg/m^3^), were lower than the national standards. No spatial cluster of PM_2.5_ concentrations existed in both 2014 and 2023 (Figure 1).

### Actual mortality of COPD and lung cancer in 2014 and 2023

The actual mortality rate of COPD in Lanzhou city raised from 39.89/100 000 persons in 2014 to 47.36/100 000 persons in 2023, of lung cancer raised from 18.86/100 000 persons in 2014 to 47.36/100 000 persons in 2023. For COPD, the actual mortality rate increased substantially in all districts/counties except Chengguan district and Yuzhong county, with a slight change in Chengguan district from 30.96/100 000 persons to 30.22/100 000 persons and kind of significant change in Yuzhong county, which dropped from 165.56/100 000 persons to 134.73/100 000 persons. As for lung cancer, the actual mortality rate increased significantly in all districts except Yuzhong county, which dropped from 25.05/100 000 persons to 19.74/100 000 persons. Both in 2014 and 2023, COPD mortality rates were the highest in the Yuzhong county, 165.56/100 000 persons in 2014 and 134.73/100 000 persons in 2023. As for lung cancer, the mortality rate is highest in Chengguan district in 2014 with 26.81/100 000 persons, and in Xigu district in 2023, with 54.07/100 000 persons (Table 2).

**Table 2.** Actual mortality of chronic obstructive pulmonary disease (COPD) and lung cancer among adults ≥25 years, Lanzhou City, China, 2014 and 2023

District/county	COPD				Lung cancer			
	2014		2023		2014		2023	
	mortality	mortality rate [n/100 000 persons/year]	mortality	mortality rate [n/100 000 persons/year]	mortality	mortality rate [n/100 000 persons/year]	mortality	mortality rate [n/100 000 persons/year]
Lanzhou	992	39.89	1422	47.36	469	18.86	985	32.80
Chengguan	283	30.96	358	30.22	245	26.81	384	32.41
Qilihe	46	11.82	139	24.94	58	14.90	142	25.48
Xigu	55	20.06	109	37.54	53	19.33	157	54.07
Anning	22	14.27	42	18.84	13	8.43	75	33.65
Honggu	13	13.03	84	78.00	10	10.02	44	40.86
Yongdeng	91	31.63	226	93.12	14	4.87	86	35.44
Gaolan	26	28.16	34	43.77	7	7.58	34	43.77
Yuzhong	456	165.56	430	134.73	69	25.05	63	19.74

### Changes of population aged ≥65 years in Lanzhou city from 2014 to 2023

**Figure 2.**
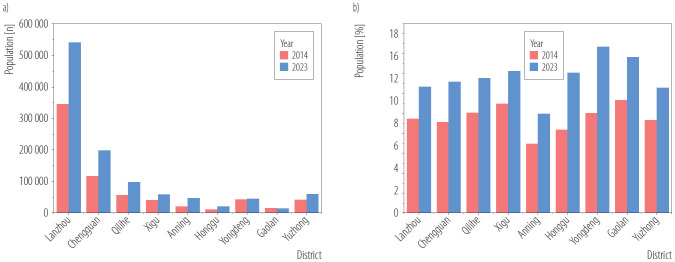
a) Number and b) proportion of people aged ≥65 years in Lanzhou city, China, 2014 and 2023

It can be seen in Figure 2 that the number of people aged ≥65 years has raised from 345 090 in 2014 to 541 200 in 2023, and the proportion has risen from 9.48% in 2014 to 12.71% in 2023 in Lanzhou city. The most significant changes in the proportion of people aged ≥65 were in Yongdeng county (10.02% in 2014 and 16.70% in 2023) and Honggu district (8.39%in 2014 and 14.09% in 2023).

### Premature mortality attributable to PM_2.5_ analysis in 2014 and 2023

#### Cause-specific premature mortality

Using the GEMM model, the results showed that the premature mortality rate of COPD attributable to PM_2.5_ were 7.4/100 000 persons (95% CI: 3.68–10.73) in 2014 and 6.36/100 000 persons (95% CI: 3.12–9.36) in 2023, respectively. These accounted for 18.55% (95% CI: 9.27–26.91%) of the total deaths of COPD in 2014 and 13.43% (95% CI: 6.61–19.76%) in 2023. The premature mortality rates of lung cancer attributable to PM_2.5_ were 4.03/100 000 persons (95% CI: 2.50–5.42) in 2014 and 4.99/100 000 persons (95% CI: 3.05–6.81) in 2023, respectively. These accounted for 21.32% (95% CI: 13.22–28.78%) of the total deaths of lung cancer in 2014 and 15.23% (95% CI: 9.24–20.71%) in 2023 (Table 3). The results of the stability test using the IER model are shown in Table 4.

**Table 3.** Premature mortality of chronic obstructive pulmonary disease (COPD) and lung cancer among adults ≥25 years, Lanzhou City, China, 2014 and 2023

Variable	COPD								Lung cancer							
	2014				2023				2014				2023			
	premature mortality	95% CI	premature mortality rate [n/100 000 persons/year]	95% CI	premature mortality	95% CI	premature mortality rate [n/100 000 persons/year]	95% CI	premature mortality	95% CI	premature mortality rate [n/100 000 persons/year]	95% CI	premature mortality	95% CI	premature mortality rate [n/100 000 persons/year]	95% CI
Lanzhou	184	92–267	7.4	3.68–10.73	191	94–281	6.36	3.12–9.36	100	62–135	4.03	2.50–5.42	150	91–204	4.99	3.05–6.81
25–44 years	1	1–2	0.1	0.05–0.14	1	0–1	0.05	0.02–0.07	3	2–3	0.22	0.14–0.30	2	1–2	0.11	0.08–0.17
45–64 years	16	8–23	1.63	0.81–2.37	13	6–19	0.92	0.45–1.36	33	20–44	3.33	2.06–4.48	33	23–52	2.41	1.71–3.82
≥65 years	167	83–242	48.32	24.04–70.10	178	87–262	33.26	16.29–48.97	65	40–87	18.77	11.63–25.25	95	67–150	17.75	12.55–28.05
Chengguan	52	26–76	5.71	2.84–8.28	48	24–71	4.06	1.99–5.98	52	32–70	5.69	3.52–7.66	59	36–80	4.94	3.02–6.74
25–44 years	0	0–0	0	0–0	0	0–1	0.07	0.04–0.11	2	1–2	0.37	0.23–0.50	1	0–1	0.1	0.07–0.15
45–64 years	5	2–7	1.4	0.70–2.03	3	2–5	0.7	0.34–1.03	16	10–22	4.77	2.95–6.42	11	8–18	2.43	1.71–3.83
≥65 years	47	24–69	40.59	20.19–58.91	45	22–66	22.44	10.99–33.04	34	21–46	29.12	18.03–39.18	39	27–61	19.56	13.83–30.91
Qilihe	9	4–13	2.24	1.12–3.25	18	9–27	3.31	1.62–4.88	13	8–17	3.26	2.02–4.38	21	13–29	3.83	2.34–5.23
25–44 years	0	0–0	0	0–0	0	0–0	0	0–0	0	0–0	0.12	0.08–0.16	0	0–0	0–0	0–0
45–64 years	1	0–1	0.37	0.18–0.54	2	1–3	0.83	0.41–1.23	3	2–4	1.71	1.06–2.30	6	4–10	2.73	1.93–4.31
≥65 years	8	4–12	14.45	7.20–20.93	17	8–24	16.96	8.30–24.98	10	6–13	17.45	10.82–23.44	12	9–19	12.6	8.90–19.91
Xigu	11	5–15	3.84	1.91–5.56	15	7–22	5.19	2.55–7.64	12	7–16	4.27	2.65–5.73	25	15–34	8.49	5.19–11.57
25–44 years	0	0–0	0.16	0.08–0.23	0	0–0	0.12	0.06–0.17	0	0–0	0	0–0	0	0–0	0.23	0.16–0.36
45–64 years	1	0–1	0.69	0.34–1.00	1	0–1	0.48	0.24–0.71	4	3–6	3.98	2.47–5.34	5	4–8	3.67	2.60–5.80
≥65 years	10	5–14	23.77	11.85–34.42	14	7–21	24.24	11.89–35.65	7	5–10	18.11	11.23–24.31	16	11–25	26.76	18.93–42.22
Anning	4	2–6	2.64	1.31–3.83	6	3–8	2.53	1.24–3.73	3	2–4	1.8	1.11–2.42	11	7–16	5.12	3.13–6.99
25–44 years	0	0–0	0	0–0	0	0–0	0	0–0	0	0–0	0	0–0	0	0–0	0.06	0.04–0.10
45–64 years	0	0–1	0.66	0.33–0.96	0	0–0	0	0–0	1	1–1	1.91	1.18–2.57	2	2–4	2.11	1.49–3.33
≥65 years	4	2–5	18.29	9.10–26.54	6	3–8	11.95	5.86–17.6	2	1–2	8.43	5.22–11.35	7	5–12	15.87	11.22–25.07
Honggu	2	1–3	2.36	1.17–3.43	11	5–16	10.08	4.93–14.86	2	1–3	2.09	1.29–2.82	6	4–9	5.97	3.64–8.15
25–44 years	0	0–0	0.38	0.19–0.55	0	0–0	0	0–0	0	0–0	0	0–0	0	0–0	0.3	0.21–0.48
45–64 years	1	0–1	1.77	0.88–2.57	1	0–1	1.06	0.52–1.57	1	1–1	2.04	1.26–2.74	2	1–3	3.37	2.38–5.34
≥65 years	1	1–2	12.71	6.31–18.46	10	5–15	50.91	24.91–75.09	1	1–2	10.97	6.79–14.78	4	3–6	18.63	13.16–29.49
Yongdeng	16	8–24	5.65	2.80–8.21	27	13–40	11.21	5.47–16.57	3	2–4	1	0.62–1.35	12	7–16	4.8	2.92–6.58
25–44 years	0	0–1	0.31	0.15–0.45	0	0–0	0	0–0	0	0–0	0	0–0	0	0–0	0.37	0.26–0.59
45–64 years	2	1–3	1.39	0.69–2.02	3	1–4	3.13	1.53–4.63	1	1–1	0.8	0.49–1.08	3	2–4	2.79	1.97–4.43
≥65 years	14	7–21	32.77	16.27–47.65	24	12–36	54.01	26.36–79.86	2	1–2	4.3	2.66–5.79	7	5–12	16.1	11.35–25.56
Gaolan	5	2–7	4.92	2.44–7.16	4	2–6	5.52	2.70–8.15	1	1–2	1.52	0.94–2.06	5	3–7	6.24	3.80–8.52
25–44 years	0	0–0	0	0–0	0	0–0	0	0–0	0	0–0	0.57	0.35–0.77	0	0–0	0.62	0.44–0.98
45–64 years	0	0–0	0.42	0.21–0.61	0	0–0	0.78	0.38–1.15	0	0–0	0.48	0.30–0.65	1	1–2	4.18	2.95–6.62
≥65 years	4	2–6	28.51	14.14–41.5	4	2–6	27.65	13.52–40.81	1	1–1	6.56	4.05–8.84	3	2–4	18.51	13.07–29.34
Yuzhong	80	40–117	29.17	14.47–42.44	52	25–77	16.34	7.98–24.16	14	9–19	5.08	3.14–6.85	9	5–12	2.7	1.64–3.69
25–44 years	0	0–1	0.29	0.15–0.43	0	0–0	0.08	0.04–0.13	0	0–1	0.34	0.21–0.46	0	0–0	0.08	0.06–0.13
45–64 years	7	3–9	5.73	2.84–8.34	3	1–4	1.85	0.900–2.73	6	4–8	5.34	3.30–7.21	2	1–3	1.45	1.03–2.31
≥65 years	73	36–107	176.19	87.42–256.33	49	24–73	83.16	40.59–122.92	7	5–10	17.98	11.11–24.25	5	4–8	8.91	6.28–14.15

**Table 4.** Premature mortality of chronic obstructive pulmonary disease (COPD) and lung among adults ≥25 years, Lanzhou City, China, 2014 and 2023, calculated by integrated exposure-response (IER model)

Variable	COPD								Lung cancer							
	2014				2023				2014				2023			
	premature mortality	95% CI	premature mortality rate [n/100 000 persons/year]	95% CI	premature mortality	95% CI	premature mortality rate [n/100 000 persons/year]	95% CI	premature mortality	95% CI	premature mortality rate [n/100 000 persons/year]	95% CI	premature mortality	95% CI	premature mortality rate [n/100 000 persons/year]	95% CI
Lanzhou	145	86–202	5.85	3.44–8.13	167	96–236	5.57	3.2–7.85	106	55–151	4.28	2.21–6.07	181	96–255	6.02	3.2–8.49
25–44 years	1	1–1	0.08	0.04–0.11	1	0–1	0.04	0.02–0.06	3	1–4	0.24	0.12–0.33	2	1–3	0.15	0.08–0.21
45–64 years	13	8–18	1.29	0.76–1.8	11	6–16	0.81	0.47–1.14	35	18–50	3.54	1.83–5.02	46	24–65	3.37	1.79–4.76
≥65 years	132	78–183	38.18	22.49–53.11	156	89–219	29.12	16.74–41.08	69	36–98	19.92	10.31–28.27	132	70–187	24.8	13.19–34.98
Chengguan	41	24–58	4.54	2.67–6.31	42	24–59	3.55	2.04–5.01	56	29–79	6.08	3.15–8.63	70	37–99	5.95	3.16–8.39
25–44 years	0	0–0	0	0–0	0	0–0	0.06	0.04–0.09	2	1–3	0.4	0.21–0.57	1	0–1	0.13	0.07–0.19
45–64 years	4	2–5	1.11	0.65–1.55	3	2–4	0.61	0.35–0.86	17	9–25	5.1	2.64–7.23	16	8–22	3.38	1.8–4.77
≥65 years	38	22–52	32.27	19.02–44.91	39	22–55	19.61	11.28–27.66	36	19–52	31.11	16.1–44.15	54	29–76	27.28	14.5–38.47
Qilihe	7	4–9	1.73	1.02–2.41	16	9–23	2.93	1.69–4.14	13	7–19	3.38	1.75–4.8	26	14–37	4.67	2.49–6.59
25–44 years	0	0–0	0	0–0	0	0–0	0	0–0	0	0–0	0.13	0.07–0.18	0	0–0	0	0–0
45–64 years	0	0–1	0.29	0.17–0.4	2	1–2	0.74	0.42–1.04	3	1–4	1.77	0.92–2.52	9	5–12	3.86	2.05–5.44
≥65 years	6	4–9	11.16	6.58–15.53	15	8–21	15.01	8.63–21.18	10	5–14	18.09	9.36–25.67	17	9–25	17.81	9.47–25.12
Xigu	8	5–11	2.94	1.73–4.09	13	7–18	4.41	2.54–6.23	12	6–17	4.39	2.27–6.22	29	15–41	9.92	5.27–13.99
25–44 years	0	0–0	0.12	0.07–0.17	0	0–0	0.1	0.06–0.14	0	0–0	0	0–0	0	0–1	0.31	0.16–0.44
45–64 years	1	0–1	0.53	0.31–0.73	1	0–1	0.41	0.23–0.58	5	2–6	4.08	2.11–5.79	7	4–10	4.97	2.64–7.01
≥65 years	7	4–10	18.2	10.72–25.32	12	7–17	20.6	11.85–29.06	7	4–11	18.6	9.62–26.39	21	11–30	36.21	19.25–51.07
Anning	3	2–4	2.09	1.23–2.91	5	3–7	2.21	1.27–3.12	3	2–4	1.91	0.99–2.72	14	7–19	6.17	3.28–8.71
25–44 years	0	0–0	0	0–0	0	0–0	0	0–0	0	0–0	0	0–0	0	0–0	0.09	0.05–0.12
45–64 years	0	0–0	0.53	0.31–0.73	0	0–0	0	0–0	1	1–2	2.04	1.05–2.89	3	2–4	2.94	1.56–4.15
65 years	3	2–4	14.49	8.54–20.17	5	3–7	10.46	6.01–14.75	2	1–3	8.98	4.64–12.74	10	6–15	22.15	11.78–31.24
Honggu	2	1–3	1.91	1.12–2.66	10	6–14	9.17	5.27–12.94	2	1–3	2.27	1.18–3.23	8	4–11	7.5	3.99–10.57
25–44 years	0	0–0	0.31	0.18–0.43	0	0–0	0	0–0	0	0–0	0	0–0	0	0–0	0.44	0.23–0.62
45–64 years	1	0–1	1.43	0.84–1.99	0	0–1	0.97	0.56–1.37	1	0–1	2.22	1.15–3.15	2	1–3	4.91	2.61–6.93
≥65 years	1	1–2	10.28	6.05–14.3	9	5–13	46.31	26.64–65.34	1	1–2	11.93	6.17–16.93	6	3–8	27.11	14.41–38.23
Yongdeng	13	8–19	4.64	2.73–6.45	27	15–37	10.95	6.3–15.44	3	2–5	1.1	0.57–1.57	16	8–22	6.5	3.46–9.17
25–44 years	0	0–0	0.25	0.15–0.35	0	0–0	0	0–0	0	0–0	0	0–0	0	0–1	0.59	0.31–0.83
45–64 years	1	1–2	1.14	0.67–1.59	3	2–4	3.06	1.76–4.31	1	1–2	0.88	0.46–1.25	4	2–6	4.38	2.33–6.17
≥65 years	12	7–16	26.91	15.85–37.44	24	14–33	52.76	30.34–74.43	2	1–3	4.75	2.46–6.74	11	6–16	25.27	13.44–35.65
Gaolan	4	2–5	4.13	2.43–5.74	4	2–6	5.14	2.96–7.26	2	1–2	1.72	0.89–2.44	6	3–9	8.03	4.27–11.33
25–44 years	0	0–0	0	0–0	0	0–0	0	0–0	0	0–0	0.64	0.33–0.91	0	0–0	0.92	0.49–1.3
45–64 years	0	0–0	0.35	0.21–0.49	0	0–0	0.73	0.42–1.03	0	0–0	0.54	0.28–0.77	2	1–3	6.24	3.32–8.8
≥65 years	4	2–5	23.9	14.08–33.26	4	2–5	25.76	14.81–36.34	1	1–2	7.4	3.83–10.5	4	2–6	27.64	14.7–38.99
Yuzhong	67	39–93	24.26	14.29–33.75	51	29–71	15.84	9.11–22.34	16	8–22	5.68	2.94–8.07	12	6–16	3.62	1.93–5.11
25–44 years	0	0–0	0.24	0.14–0.34	0	0–0	0.08	0.05–0.12	0	0–1	0.38	0.2–0.54	0	0–0	0.13	0.07–0.18
45–64 years	5	3–8	4.77	2.81–6.63	2	1–3	1.79	1.03–2.53	7	4–10	5.98	3.1–8.49	3	2–4	2.27	1.2–3.19
≥65 years	61	36–85	146.53	86.33–203.87	48	28–68	80.59	46.35–113.69	8	4–12	20.13	10.41–28.57	8	4–12	13.87	7.38–19.57

#### Region-specific premature mortality

**Figure 3.**
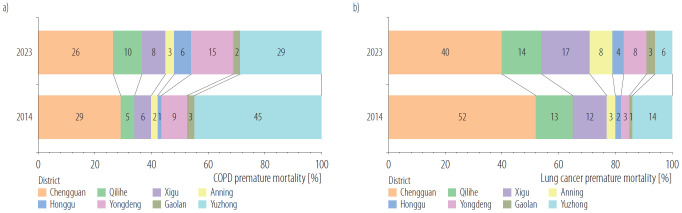
Proportion of a) chronic obstructive pulmonary disease (COPD) and b) lung cancer premature mortality by district in Lanzhou city, China, 2014 and 2023

As showed in Figure 3, for COPD, Yuzhong County accounted for the largest proportion of premature mortality (45% in 2014 and 29% in 2023), while Gaolan County had the smallest proportion (3% in 2014 and 2% in 2023). For lung cancer, Chengguan District represented the largest share of premature mortality (52% in 2014 and 40% in 2023), whereas Gaolan County again showed the smallest proportion (1% in 2014 and 3% in 2023).

#### Age-specific premature mortality

**Figure 4.**
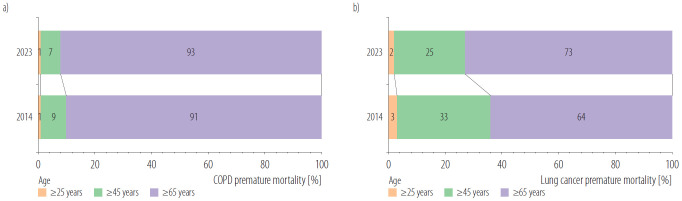
Proportion of premature mortality from a) chronic obstructive pulmonary disease (COPD) and b) lung cancer by age group in Lanzhou city, China, 2014 and 2023

Among each age group, premature mortalities attributable to PM_2.5_ in the ≥65-year age group are all much higher than the other 2 age groups, both in COPD and lung cancer in 2014 and 2023. Furthermore, the proportion of total premature deaths accounted for by the ≥65-year group increased from 2014 to 2023. For COPD, the proportion of premature mortality rose from 91% to 93%, while for lung cancer, it increased more markedly from 64% to 73% as illustrated in Figure 4.

#### Simulation of mortality benefits of lower PM_2.5_

**Figure 5.**
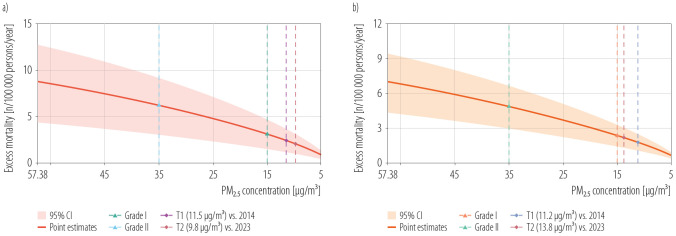
Changes in a) COPD and b) lung cancer premature mortality in Lanzhou city, China, 2014 and 2023

In Figures 5, the T1 and T2 thresholds represent the PM_2.5_ concentrations at which premature mortality becomes significantly lower than the 2014 and 2023 baseline levels, respectively. The simulation was conducted using the 2023 population and actual mortality data, with PM_2.5_ concentration as the sole variable. The PM_2.5_ concentration starts to decline from 57.38 μg/m^3^(PM_2.5_ concentration in 2014 in Lanzhou city), resulting in a more significant reduction in premature mortality when the decline reaches 9.80 μg/m^3^ in COPD deaths and 13.80 μg/m^3^ in lung cancer deaths compared with the situation in 2023 (T2 in Figure 5a and 5b). While comparing with the situation in 2014, a more significant reduction would be seen when the decline reaches 11.50 μg/m^3^ in COPD and 11.20 μg/m^3^ in lung cancer (T1 in Figure 5a and 5b). The PM_2.5_-attributable mortality is also estimated for 2 key benchmarks: the national Grade II standard (35 μg/m³) and the national Grade I standard (15 μg/m³).

T1 and T2 thresholds represent the PM_2.5_ concentrations at which premature mortality becomes significantly lower than the 2014 and 2023 baseline levels.

## DISCUSSION

The study showed that the average concentration of PM_2.5_ was decreased significantly from 2014 to 2023 in Lanzhou City; however, there is no change in premature mortality of COPD and lung cancer attributable to PM_2.5_, which is also more in line with a previous study, where it has been found that while policies have reduced ambient pollutant concentrations, the health burden continues to rise in China [24]. Globally, the number of COPD deaths attributable to PM_2.5_ increased by 90% from 1990 to 2019, most occurred in middle sociodemographic index (SDI) region [8].

Premature mortality is influenced by PM_2.5_ concentration, baseline mortality and resident population. From 2014 to 2023, the average PM_2.5_ concentration in Lanzhou City decreased from 57.38 μg/m³ to 36.08 μg/m³, indicating the effect of air quality policies, though it remained above the national Grade II standard (The annual average value shall not be higher than 35μg/m^3^ in residential areas, mixed commercial-transportation residential areas, cultural areas, industrial areas and rural areas) [25]. Spatially, concentrations were highest in the 4 main urban districts and decreased toward the periphery, due to factors such as their higher population density, industrial activity, and traffic volume. Consistent with studies identifying densely populated areas as high-risk zones [26], the health risk of PM_2.5_ was concentrated in these urban cores. In 2023, these 4 urban districts contained 75.12% of the city's population. Accordingly, they accounted for a disproportionate share of PM_2.5_-attributable deaths: 45.57% for COPD and 76.95% for lung cancer, highlighting clear intra-city disparities.

The imperative for sustained air quality management is not confined to China. Recent evidence from Europe underscores that this is a pervasive global challenge. According to the Europe's Air Quality Status 2023 report, despite long-term reductions in emissions, 97% of the EU's urban population in 2021 remained exposed to fine particulate matter (PM_2.5_) levels exceeding the stringent WHO 2021 annual guideline of 5 µg/m³ [27]. This widespread exceedance of health-based thresholds in a highly regulated region highlights the immense difficulty in achieving safe air quality levels, even with advanced policies and technologies. This situation illustrates that reducing emissions does not automatically translate into immediate health gains at the population level, due to factors like population aging and legacy exposure. The authors' findings of significant disease burdens in China, when viewed alongside this European evidence, reinforce a critical public health message: continuous and intensified efforts to reduce air pollution are urgently needed worldwide to mitigate the substantial and ongoing burden of respiratory and cardiovascular diseases. This global perspective aligns with the objectives of international policy frameworks such as the WHO 2021 Global Air Quality Guidelines and the EU's Zero Pollution Action Plan, which call for persistent action to bridge the gap between current exposures and health-protective targets.

According to this study, both the population size and the actual number of deaths from COPD and lung cancer increased notably over the past 10 years in Lanzhou City, a trend consistent with some global studies [28,29]. This increase occurred alongside population aging, as evidenced by the rise in the proportion of people aged ≥65 years from 9.48% in 2014 to 12.71% in 2023, which contributed to the relatively high baseline mortality rates. It has been found that among LC deaths attributable to PM_2.5_, population aging accounted for 43.0% of the increase in mortality. Regarding PM_2.5_-related COPD deaths, aging corresponded to an increase of 18.547/100 000 population [30]. The mortality rate attributable to PM_2.5_ showed substantial disparities among different age groups, with a particularly high and increasing burden in the ≥65-year age group. Specifically, the proportion of premature mortality among those aged ≥65 year increased from 91% to 93% for COPD, and from 64% to 73% for lung cancer between 2014 and 2023. It indicated that older people were more susceptible to the adverse effects of air pollution, and the age structure should be taken into account when estimating the premature mortality attributable to PM_2.5_.

The substantial health burden in China underscores the need for stringent air quality standards. National estimates show that *per capita* PM_2.5_-related mortality reaches 95/100 000 person-years, and achieving the WHO interim target (35 μg/m³) would reduce premature mortality by only 12.6%, whereas a stricter standard (10 μg/m³) could prevent 73.0% of deaths [31]. The simulation results of this study suggest that, assuming the baseline mortality rate and population size remain unchanged, a reduction in PM_2.5_ concentration to 9.8 μg/m³ or 13.80 μg/m^3^ would be required to achieve a significant decline in the attributable mortality rates of COPD and lung cancer. However, from a practical perspective, it is highly challenging for PM_2.5_ concentrations to reach this level under current governance conditions in a short period of time.

It is worth noticing that there are many other factors that influence mortality from these diseases, like smoking [32], other pollutants – such as sulfur dioxide, nitrogen dioxide, and ozone, type of treatment, population density [31], comorbidities [33,34] and socioeconomic status [35,36]. Premature prevention for lung cancer (tobacco control) is considered to be the main reason for the decline in premature mortality rates from lung cancer in most countries [37], and smoking cessation also lowers mortality in COPD patients [38,39]. In Gansu province, the prevalence of smoking among the male population is 56.7%, which is one of the highest levels in China [40]. Therefore, in addition to reducing PM_2.5_ pollution levels, it is important to focus on improving healthcare and promoting healthier lifestyles to lower the baseline mortality rate and reduce the numbers of PM_2.5_-related death in the future.

In summary, the differential trends in PM_2.5_-attributable mortality for COPD and lung cancer underscore the necessity for disease-specific public health strategies. Furthermore, mitigating the overall burden requires tackling the inter-district inequalities in economic development and healthcare access that currently exacerbate population vulnerability.

### Limitations

This study has several limitations. First, the GEMM model, while robust, was developed from global cohorts, and its risk estimates may not be fully transferable to the local population in Lanzhou due to differences in underlying health status and environmental factors. Second, the propagation of uncertainty, though attempted, may not be comprehensive, as the authors primarily focused on the uncertainty from the concentration-response functions, with less emphasis on potential errors in baseline mortality and exposure data. Third, exposure measurement error is inevitable, as the PM_2.5_ concentrations were derived from the National Tibetan Plateau Science Data Center, which may not capture fine-scale spatial variability or personal exposure accurately. Fourth, potential misclassification of cause of death, particularly between COPD and other respiratory diseases, in the vital registration system could affect the accuracy of baseline mortality rates. Additionally, the analysis was restricted to COPD and lung cancer, excluding other PM_2.5_-related diseases. Finally, the GEMM model does not account for spatial spillover effects of air pollution. Despite these limitations, the consistent findings using an alternative model (IER) in the authors' sensitivity analysis strengthen the robustness of their primary conclusions regarding the substantial disease burden attributable to PM_2.5_ in Lanzhou.

## CONCLUSIONS

In conclusion, despite improved air quality in Lanzhou in 2014–2023, PM_2.5_-attributable premature mortality from COPD and lung cancer showed no significant decline, largely due to population aging and increased baseline mortality. Substantial regional disparities were observed, with some areas experiencing increased mortality despite meeting air quality standards, while the elderly population consistently bore the highest burden. It is also critical to note that due to the multi-year development cycles of these chronic diseases, the positive impact of air quality policies on mortality rates may only become apparent over a longer observation period. These findings underscore the need for targeted and forward-looking public health interventions that address both air quality and demographic challenges to achieve meaningful health benefits.

## References

[1] GBD 2019 Risk Factors Collaborators. Global burden of 87 risk factors in 204 countries and territories, 1990–2019: a systematic analysis for the Global Burden of Disease Study 2019. Lancet. 2020;396(10258):1223–49. 10.1016/S0140-6736(20)30752-2. 33069327 PMC7566194

[2] GBD 2021 Risk Factors Collaborators. Global burden and strength of evidence for 88 risk factors in 204 countries and 811 subnational locations, 1990–2021: a systematic analysis for the Global Burden of Disease Study 2021. Lancet. 2024;403(10440):2162–203. 10.1016/S0140-6736(24)00933-4. 38762324 PMC11120204

[3] European Environment Agency [Internet]. Copenhagen: The Agency; 2025 [cited 2025 Dec 16]. Premature deaths due to exposure to fine particulate matter in Europe. Available from: https://www.eea.europa.eu/en/analysis/indicators/health-impacts-of-exposure-to.

[4] Yousefi R, Shaheen A, Wang F, Ge Q, Wu R, Lelieveld J, et al. Fine particulate matter (PM_2.5_) trends from land surface changes and air pollution policies in China during 1980–2020. J Environ Manage. 2023;326:116847. 10.1016/j.jenvman.2022.116847. 36436250

[5] Zheng S, Schlink U, Ho KF, Singh RP, Pozzer A. Spatial distribution of PM_2.5_-related premature mortality in China. Geohealth. 2021;5(10):e2021GH000532. 10.1029/2021GH000532. PMC8647684 34926970

[6] World Health Organization [Internet]. Geneva: The Organization; 2024 [cited 2025 Dec 16]. Sustainable Development Goal indicator 3.9.1: mortality attributed to air pollution. Available from: https://www.who.int/publications/i/item/9789240099142.

[7] Halpin DMG. Mortality of patients with COPD. Expert Rev Respir Med. 2024;18(6):381–95. 10.1080/17476348.2024.2375416. 39078244

[8] Yang X, Zhang T, Zhang Y, Chen H, Sang S. Global burden of COPD attributable to ambient PM_2.5_ in 204 countries and territories, 1990 to 2019: a systematic analysis for the Global Burden of Disease Study 2019. Sci Total Environ. 2021;796:148819. 10.1016/j.scitotenv.2021.148819. 34265615

[9] Sin DD, Doiron D, Agusti A, Anzueto A, Barnes PJ, Celli BR, et al. Air pollution and COPD: GOLD 2023 committee report. Eur Respir J. 2023;61(5):2302469. 10.1183/13993003.02469-2022. 36958741

[10] Bray F, Laversanne M, Sung H, Ferlay J, Siegel RL, Soerjomataram I, et al. Global cancer statistics 2022: GLOBOCAN estimates of incidence and mortality worldwide for 36 cancers in 185 countries. CA Cancer J Clin. 2024;74(3):229–63. 10.3322/caac.21834. 38572751

[11] Guo H, Li W, Wu J. Ambient PM_2.5_ and annual lung cancer incidence: a nationwide study in 295 Chinese counties. Int J Environ Res Public Health. 2020;17(5):1481. 10.3390/ijerph17051481. 32106556 PMC7084498

[12] Cao W, Chen HD, Yu YW, Li N, Chen WQ. Changing profiles of cancer burden worldwide and in China: a secondary analysis of the global cancer statistics 2020. Chin Med J (Engl). 2021;134(7):783–91. 10.1097/CM9.0000000000001474. 33734139 PMC8104205

[13] Kuzma L, Kurasz A, Dabrowski EJ, Dobrzycki S, Bachórzewska-Gajewska H. Short-term effects of “Polish smog” on cardiovascular mortality in the green lungs of Poland: a case-crossover study with 4,500,000 person-years (PL-PARTICLES study). Atmosphere (Basel). 2021;12(10):1270. 10.3390/atmos12101270.

[14] Kuźma Ł, Struniawski K, Pogorzelski S, Bachórzewska-Gajewska H, Dobrzycki S. Gender differences in association between air pollution and daily mortality in the capital of the green lungs of Poland–population-based study with 2,953,000 person-years of follow-up. J Clin Med. 2020;9(8):2351. 10.3390/jcm9082351. 32717977 PMC7464921

[15] Hou T, Zhu L, Wang Y, Peng L. Oxidative stress is the pivot for PM_2.5_-induced lung injury. Food Chem Toxicol. 2024;184:114362. 10.1016/j.fct.2023.114362. 38101601

[16] Noh M, Sim JY, Kim J, et al. Particulate matter-induced metabolic recoding of epigenetics in macrophages drives pathogenesis of chronic obstructive pulmonary disease. J Hazard Mater. 2024;464:132932. 10.1016/j.jhazmat.2023.132932. 37988864

[17] Afthab M, Hambo S, Kim H, Alhamad A, Harb H. Particulate matter-induced epigenetic modifications and lung complications. Eur Respir Rev. 2024;33(172):240129. 10.1183/16000617.0129-2024. 39537244 PMC11558539

[18] Liao Q, Li Z, Li Y, Dai X, Kang N, Niu Y, et al. Specific analysis of PM_2.5_-attributed disease burden in typical areas of Northwest China. Front Public Health. 2023;11:1338305. 10.3389/fpubh.2023.1338305. 38192558 PMC10771959

[19] Zhang M, Jia J, Wang B, Zhang W, Gu C, Zhang X, et al. Source apportionment of fine particulate matter during the day and night in Lanzhou, NW China. Int J Environ Res Public Health. 2022;19(12):7091. 10.3390/ijerph19127091. 35742335 PMC9222658

[20] Dong J, Liu Y, Bao H. Revalue associations of short-term exposure to air pollution with respiratory hospital admissions in Lanzhou, China after the control and treatment of current pollution. Int J Hyg Environ Health. 2021;231:113658. 10.1016/j.ijheh.2020.113658. 33166757

[21] Burnett R, Chen H, Szyszkowicz M, Fann N, Hubbell B, Pope CA 3rd, et al. Global estimates of mortality associated with long-term exposure to outdoor fine particulate matter. Proc Natl Acad Sci U S A. 2018;115(38):9592–7. 10.1073/pnas.1803222115. 30181279 PMC6156628

[22] Xue T, Zhu T, Zheng Y, Liu J, Li X, Zhang Q. Change in the number of PM_2.5_-attributed deaths in China from 2000 to 2010: comparison between estimations from census-based epidemiology and pre-established exposure-response functions. Environ Int. 2019;129:430–7. 10.1016/j.envint.2019.05.067. 31154145

[23] Chung CY, Yang J, Yang X, Jun H. Mathematical modeling in the health risk assessment of air pollution-related disease burden in China: a review. Front Public Health. 2022;10:1060153. 10.3389/fpubh.2022.1060153. 36504933 PMC9727382

[24] Liu M, Saari RK, Zhou G, Li J, Han L, Liu X. Recent trends in premature mortality and health disparities attributable to ambient PM_2.5_ exposure in China: 2005–2017. Environ Pollut. 2021;279:116882. 10.1016/j.envpol.2021.116882. 33756244

[25] Standards Press of China. Ambient air quality standards: GB 3095-2012. Beijing: Standards Press of China; 2012.

[26] Zhang Z, Shao C, Guan Y, et al. Socioeconomic factors and regional differences of PM_2.5_ health risks in China. J Environ Manage. 2019;251:109564. 10.1016/j.jenvman.2019.109564. 31557670

[27] European Environment Agency [Internet]. Copenhagen: The Agency; 2023 [cited 2025 Dec 16]. Europe's air quality status 2023. Available from: https://www.eea.europa.eu/en/analysis/publications/europes-air-quality-status-2023

[28] Li X, Cao X, Guo M, Xie M, Liu X. Trends and risk factors of mortality and disability adjusted life years for chronic respiratory diseases from 1990 to 2017: systematic analysis for the Global Burden of Disease Study 2017. BMJ. 2020;368:m234. 10.1136/bmj.m234. 32075787 PMC7190065

[29] Zhou J, Xu Y, Liu J, Feng L, Yu J, Chen D. Global burden of lung cancer in 2022 and projections to 2050: incidence and mortality estimates from GLOBOCAN. Cancer Epidemiol. 2024;93:102693. 10.1016/j.canep.2024.102693. 39536404

[30] Liu X, Zhou H, Yi X, Zhang X, Lu Y, Zhou W, et al. Decomposition analysis of lung cancer and COPD mortality attributable to ambient PM_2.5_ in China (1990-2021). Asia Pac J Oncol Nurs. 2025;12(2):100653. 10.1016/j.apjon.2025.100653. 40026876 PMC11869952

[31] Li Y, Zhao X, Liao Q, Tao Y, Bai Y. Specific differences and responses to reductions for premature mortality attributable to ambient PM_2.5_ in China. Sci Total Environ. 2020;742:140643. 10.1016/j.scitotenv.2020.140643. 32640394

[32] Yang X, Man J, Chen H, Zhang T, Yin X, He Q, et al. Temporal trends of the lung cancer mortality attributable to smoking from 1990 to 2017: a global, regional and national analysis. Lung Cancer. 2021;152:49–57. 10.1016/j.lungcan.2020.12.007. 33348250

[33] Gould MK, Munoz-Plaza CE, Hahn EE, Lee JS, Parry C, Shen E. Comorbidity profiles and their effect on treatment selection and survival among patients with lung cancer. Ann Am Thorac Soc. 2017;14(10):1571–80. 10.1513/AnnalsATS.201701-030OC. 28541748

[34] Divo M, Cote C, de Torres JP, Casanova C, Marin JM, Pinto-Plata V, et al. Comorbidities and risk of mortality in patients with chronic obstructive pulmonary disease. Am J Respir Crit Care Med. 2012;186(2):155–61. 10.1164/rccm.201201-0034OC. 22561964

[35] Song S, Duan Y, Huang J, Wong MCS, Chen H, Trisonlini MG, et al. Socioeconomic inequalities in premature cancer mortality among U.S. counties during 1999 to 2018. Cancer Epidemiol Biomarkers Prev. 2021;30(7):1375–86. 10.1158/1055-9965.EPI-20-1534. 33947656

[36] Álvarez-Aceves M, Hernández-Ávila JE. Premature mortality and socioeconomic inequalities in Mexico. Lancet Public Health. 2023;8(8):e660–1. 10.1016/S2468-2667(23)00177-9. 37633671

[37] Murthy SS, Trapani D, Cao B, et al. Premature mortality trends in 183 countries by cancer type, sex, WHO region, and World Bank income level in 2000–19: a retrospective, cross-sectional, population-based study. Lancet Oncol. 2024;25(7):969–78. 10.1016/S1470-2045(24)00274-2. 38964357 PMC11329430

[38] Russo P, Milani F, De Iure A, Proietti S, Limonggi D, Prezioso C, et al. Effect of cigarette smoking on clinical and molecular endpoints in COPD patients. Int J Mol Sci. 2024;25(11):5834. 10.3390/ijms25115834. 38892022 PMC11172087

[39] Doo JH, Kim SM, Park YJ, Kim KH, Oh YH, Kim JS, et al. Smoking cessation after diagnosis of COPD is associated with lower all-cause and cause-specific mortality: a nationwide population-based cohort study of South Korean men. BMC Pulm Med. 2023;23(1):237. 10.1186/s12890-023-02533-1. 37394482 PMC10316560

[40] Zhang M, Yang L, Wang L, Jiang Y, Huang Z, Zhao Z, et al. Trends in smoking prevalence in urban and rural China, 2007 to 2018: findings from 5 consecutive nationally representative cross-sectional surveys. PLoS Med. 2022;19(8):e1004064. 10.1371/journal.pmed.1004064. 36006870 PMC9409540

